# The interaction between predation risk and food ration on behavior and morphology of Eurasian perch

**DOI:** 10.1002/ece3.3330

**Published:** 2017-09-14

**Authors:** Richard Svanbäck, Yinghua Zha, Christer Brönmark, Frank Johansson

**Affiliations:** ^1^ Animal Ecology Department of Ecology and Genetics Uppsala University Uppsala Sweden; ^2^ Limnology Department of Ecology and Genetics Uppsala University Uppsala Sweden; ^3^ Aquatic Ecology Department of Biology Lund University Lund Sweden

**Keywords:** body shape, environmental conditions, growth, phenotypic plasticity, predator‐induced

## Abstract

The risk of both predation and food level has been shown to affect phenotypic development of organisms. However, these two factors also influence animal behavior that in turn may influence phenotypic development. Hence, it might be difficult to disentangle the behavioral effect from the predator or resource‐level effects. This is because the presence of predators and high resource levels usually results in a lower activity, which in turn affects energy expenditure that is used for development and growth. It is therefore necessary to study how behavior interacts with changes in body shape with regard to resource density and predators. Here, we use the classic predator‐induced morphological defense in fish to study the interaction between predator cues, resource availability, and behavioral activity with the aim to determine their relative contribution to changes in body shape. We show that all three variables, the presence of a predator, food level, and activity, both additively and interactively, affected the body shape of perch. In general, the presence of predators, lower swimming activity, and higher food levels induced a deep body shape, with predation and behavior having similar effect and food treatment the smallest effect. The shape changes seemed to be mediated by changes in growth rate as body condition showed a similar effect as shape with regard to food‐level and predator treatments. Our results suggests that shape changes in animals to one environmental factor, for example, predation risk, can be context dependent, and depend on food levels or behavioral responses. Theoretical and empirical studies should further explore how this context dependence affects fitness components such as resource gain and mortality and their implications for population dynamics.

## 1 INTRODUCTION

Phenotypic plasticity is the ability of a single genotype to express different phenotypes in response to dissimilar environmental conditions (Newman, 1992; Scheiner, 1993). Phenotypic plasticity can be expressed in different traits, such as behavior, morphology, life history, and physiology. Phenotypic plasticity is favored in variable environments (DeWitt & Scheiner, 2004) and usually there are many niche dimensions such as temperature, resource levels, and predation risk that vary from one environment to another. Thus, expression of a plastic trait may be affected by more than one environmental variable. Hence, studying how plastic traits are affected by the interaction of multiple environmental factors is important for an adaptive and mechanistic understanding of the evolution of phenotypic plasticity.

Body shape is an important phenotypic trait that is plastic in many animal species. For example, many organisms adaptively change body morphology in response to predation threat (Arnqvist & Johansson, 1998; Boersma, Spaak, & De Meester, 1998; Brönmark & Miner, 1992; Krist, 2002). Changes in body morphology may affect an organism's performance in, for example, fast start, maneuverability, speed, and endurance (Benkman & Lindholm, 1991; Dayton, Saenz, Baum, Langerhans, & DeWitt, 2005; Losos, 1990; Svanbäck & Eklöv, 2003). The changes may also incur a cost through higher energy expenditures and this may, in turn, affect foraging behavior, diet choice, and predator defense (Benkman & Lindholm, 1991; Dayton et al., 2005; Losos, 1990; Svanbäck & Eklöv, 2003). A well‐studied example of phenotypically plastic body shape is provided by the crucian carp (*Carassius carassius*) where chemical cues from piscivorous fish induce a deeper body shape (Brönmark & Miner, 1992). The induced deeper body results in lower capture success by the predator which suggests that the induced deeper body is adaptive (Nilsson, Brönmark, & Pettersson, 1995). On the other hand, a deeper body is energetically costly which explains why not all fish show a deeper body shape (Brönmark & Miner, 1992; Pettersson & Brönmark, 1997).

However, in addition to predator threat, resource levels seem also to influence body shape. For example, Brönmark and Miner (1992) showed that crucian carp developed a deeper body at high resource levels compared to low resource levels, and Chivers, Zhao, Brown, Marchant, and Ferrari (2008) showed the same pattern in the closely related goldfish (*C. auratus*), possibly an effect of a higher growth rate at high resource levels (Vollestad, Varreng, & Poleo, 2004). Thus, organisms may be constrained in their morphological response due to a trade‐off with other environmental variables (Björklund & Merila, 1993; Stauffer & Gray, 2004). Further, phenotypic plasticity can be constrained by resource availability if there is a production cost of the plastic phenotype (Olsson, Svanbäck, & Eklöv, 2006). To the best of our knowledge, the combined effect of both predator threat and resources on body shape has been studied only in a few study systems: fish (Brönmark & Miner, 1992; Chivers et al., 2008; Eklöv & Jonsson, 2007; Olsson et al., 2006) and frogs (Relyea, 2004; Steiner, 2007). Nevertheless, these few examples suggest that multiple drivers of plasticity in body shape may be a common phenomenon. Furthermore, as predators usually decrease the abundance of its prey and thereby increase the *per capita* resources for the remaining prey, there is likely a combined effect of predator presence and resource abundance on prey phenotypic responses in natural populations (Holopainen, Aho, Vornanen, & Huuskonen, 1997; Tonn, Holopainen, & Paszkowski, 1994). Hence, the interaction between predator risk and resource level is probably common in nature, but we lack a thorough understanding at a mechanistic level.

Presence of predators may also change the behavior of prey (Eklöv & Persson, 1996; Teplitsky & Laurila, 2007) and it has been suggested that, at least in some cases, the predator‐induced changes in body shape could be an indirect effect mediated by behavioral changes (Bourdeau & Johansson, 2012). For example, in the presence of predators prey may reduce their activity and thus reduce their metabolic cost, and this may save energy (Holopainen et al., 1997; Scheiner & Berrigan, 1998; Steiner & Van Buskirk, 2009) that could be used to increase growth rate and ultimately affect body shape. Thus, a higher growth rate induced by either lower activity or higher resource densities may result in a similar body shape as that induced by predators (Bourdeau & Johansson, 2012). However, it is difficult to disentangle the behavioral effect from the predator or resource‐level effects as the presence of predators and high resource levels usually results in a lower activity (Andersson, Johansson, & Söderlund, 2006), and it is therefore necessary to study how behavior interacts with resource density and predator presence in order to understand the mechanisms behind changes in body shape.

Here, we studied the relative contribution of predator cues, resource availability, and activity on changes in body shape. We used Eurasian perch (*Perca fluviatilis*) as prey and Northern pike (*Esox lucius*) as predators as earlier studies have shown that perch body morphology is affected by predator cues from pike (Eklöv & Jonsson, 2007) and resource availability (Olsson et al., 2006). We manipulate resource densities and presence of pike in a factorial design while simultaneously quantifying behavior of perch. We predict that the three factors simultaneously affect and interact to influence perch body shape but that there is a hierarchy to environmental factors (i.e., predation, resource level, and behavior) that affect phenotypic plasticity (e.g., body depth). If changing body shape is costly (Olsson, Svanbäck, & Eklöv, 2007; Olsson et al., 2006), then we expect the response to the presence of predators to be depending on resource level.

## 2 MATERIALS AND METHODS

One‐year‐old perch were caught at the end of May 2013 by cast nets outside the littoral zone of Lake Mälaren (N59°20′, E17°52′), whereas pike (341.6 ± 49.2 mm, 207.3 ± 76.7 g, mean fish length and weight ± SD) were collected with fyke nets and minnow traps from Lake Messormen and Lake Hersjön in May and July 2013. Both perch and pike were allowed to acclimatize to laboratory conditions for at least 6 weeks before being used in the experiment. The experiment was carried out in 36 visually isolated 105‐L aquaria (75 × 40 × 35 cm, length × width × height) with a 3‐cm layer of fine sand on the bottom. Each aquarium was separated into two equal parts with a transparent plastic wall with holes that allowed water circulation. This setup allowed the perch to be affected by both visual and olfactory predator cues. Each aquarium had its own internal water pump and filter. Fifty percent of the water was replaced once a week. The photoperiod was 12‐h light/12‐h dark and the temperature was kept between 19 and 20°C using a thermostat heater in each aquarium.

In order to investigate the effects of food level and predator presence on morphological plasticity, we set up a 3 × 2 fully factorial experiment with three different levels of food supply, two predator treatments (presence, absence), and six replicates per treatment combination. Perch were fed frozen chironomids every day and the treatment with the highest food level received an amount equaling 15% of the initial fish body weight per day, which is close to maximum food conversion of perch at the specific size and temperature (Lessmark, 1983). The medium and low food treatments received 10% and 5% of the initial body weight, respectively. In the predator treatment, a pike was placed in the left compartment of the divided aquaria. Pike were fed juvenile perch twice every week and all pike were actively feeding during the experiment.

We put four haphazardly chosen perch (79.3 ± 4.5 mm, 4.4 ± 1.0 g, mean fish length and weight ± *SD*) in each aquarium. The experiment was run for 10 weeks to allow growth and changes in morphology in perch. One week into the experiment, one fish per aquarium was sacrificed (for another project) leaving three fish per aquarium. Because of mortality in some of the aquaria, we were left with one to three fish per aquarium (total 82 individuals, average 2.3 fish per replicate) at the end of the experiment. However, mortality was independent of treatment (ANOVA: predator; *F*_1,30_ = 0.889, *p* = .353, food; *F*_2,30_ = 1.3889, *p* = .265, predator × food; *F*_2,30_ = 0.389, *p* = .681). The amount of food supplied was adjusted according to the number of live fish per aquarium so that the treatment food ration according to body weight was kept within each replicate. The day before the termination of the experiment, the fish were fed at the intermediate food level to avoid morphological differences due to gut fullness. At the termination of the experiment, all fish were killed using an overdose of benzocaine, individually weighed (to the nearest 0.1 g), and measured for length (total length to the nearest mm). We estimated condition of each individual by first performing a regression on all log‐transformed lengths and weights and saving the residuals. From this regression, we calculated the weight for an average individual of 90 mm (the average length at the end of the experiment was 90.6 mm). Condition for each fish was then estimated as the calculated average weight plus the residual. The condition index was then used as a measure of condition in all analyses. This condition index is perfectly correlated (*r* = 1) to the residuals and also to condition index accounting for the allometric relationship between length and weight in the population. Therefore, our method to calculate condition index does not affect any statistical analyses as it keeps the order and distance among samples, that is, we would get the same statistical results as we would get using residuals. However, it is more intuitive than the traditionally used residuals as it yields units of estimated weights for a given length (Persson, Byström, & Wahlström, 2000). After length and weight measurements, the fish were placed on a piece of styrofoam, fins fixed with needles, and photographed with a digital camera.

### 2.1 Behavior

All experimental aquaria were filmed for behavioral measurements 1 week into the experiment and the day before termination of the experiment. We estimated three behaviors: distance from bottom, distance from predator, and swimming activity. These three behavioral responses are well known to be affected by both predation and resource level in fish in general and in previous studies also specifically in perch (Eklöv & Persson, 1995; Eklöv & Svanbäck, 2006; Olsson et al., 2007). The recordings were made in the morning before feeding the perch to standardize hunger levels and with the same observer every time. The pike were also not fed before the recordings to avoid kairomones from attacked and eaten conspecifics to trigger a stronger behavioral response. Each aquarium had a 5 × 5 cm grid pattern drawn on the front glass to facilitate quantification of behaviors. Using the grids, we recorded the position of each individual perch every 5 s for 1 min. From this, we calculated the average distance of the individual from the bottom of the aquaria as well as the average distance from the partition between compartments. As an estimate of activity, we measured the total distance the fish had swum during the minute of recording. We later used the activity levels at the beginning and the end of the experiment to investigate their effects on shape.

### 2.2 Body shape

Body shape was analyzed using landmark‐based geometric morphometrics (Zelditch, Swiderski, Sheets, & Fink, 2004). Sixteen homogenous landmarks on the left side of each fish (Figure 3a) were digitalized and the x and y coordinates of these landmarks were captured using TpsDIG32 (freeware, http://life.bio.sunysb.edu/morph/). All further shape analyses and visualizations were performed in R 3.0.2 (R Development Core Team 2013) using the packages “geomorph” and “shapes” (Adams & Otarola‐Castillo, 2013; Dryden, 2015). The imported individuals’ landmarks were superimposed (Procrustes superimposition) to a common coordinate system by keeping their position, size, and orientation constant. The superimposed landmarks were then used in a principal component analysis to produce principle component axes describing the shape variation. Furthermore, we used the individuals’ centroid size as a covariate in analyses of shape.

### 2.3 Statistics

All statistical analyses were performed in R 3.0.2 (R Development Core Team 2013). We used nested ANOVAs to analyze the effect of our treatments (predator and food) on length, condition, and behavior (distance from bottom, distance from predator, and swimming activity), where individuals were nested in tanks. In case of significant results, we performed post hoc tests using the package “lsmeans” with Tukey method for *p*‐value adjustment. Because of the mortality, our nested design was unbalanced. We choose not to not account for the unbalanced design as this is more conservative (less likely to be significant) (McDonald, 2014). To analyze effects on body shape, we performed MANCOVAs and subsequent ANCOVAs (with activity levels at the start and at the end of the experiment as well as centroid size and condition as covariates) based on averages from each tank to test for effects of treatments on shape (PC scores). We used activity at both the start and the end in this model as activity changed during the course of the experiment and activity at both the start and the end may influence perch body shape. To avoid colinearity among our predictor variables, we first tested for excessive correlations (*r* > 0.8) among our explanatory variables; however, we found only weak correlations among our explanatory variables (all *r* < 0.30). Because of limitations in replication, we could not use all 32 principal component axes in the MANCOVA. Our MANCOVA was instead performed with the first four principal component axes (representing 64.4% of the total variation). This decision was based on a scree plot of eigenvalues (all factors until a break in the graph was included). As we could not connect individual activity to shape from the videos, we used tank averages of activities and shape when calculating the correlation between shape and activity. For the effect of treatments and condition, we used individual‐level variation in shape for the illustrations. In all tests, we checked whether residuals deviated from normality.

To rule out that mortality affected our response variables, we added mortality to all our models (using tank averages). However, we did not find any effects of mortality on our response variables, all *p*‐values were larger than .18 except for length (*p* = .08) and PC2 (*p* = .09), and hence, we subsequently excluded mortality from our models.

To examine whether a plastic response in body shape to predation was limited by food level, we performed separate MANOVAs for each food‐level treatment and estimated the multivariate effect size of predation as 1 − λ (Sharma, 1996). This statistic should be interpreted similar to a univariate eta square and ranges from 0 to 1, where 0 indicates no relationship between the factor and the dependent variable, while 1 indicates the strongest possible relationship.

Finally, we estimated the relative overall effect sizes (% of variation explained) on shape in perch in the experiment. We used the calculated PC axes as quantitative axes summarizing among individual variation in morphology. For each PC axis, we then used an ANCOVA framework to estimate the percent variation explained by our treatments (food, predator, and the interaction food × predator), size (centroid), condition, as well as our activity measurements (swimming speed; both after 1 week and at the end of the experiment). The effect sizes were then calculated by summing the percent variation explained by each factor, across all 32 PC axes, weighted by the % of total variation attributed to each PC axis.

## 3 RESULTS

### 3.1 Condition

**Figure 1:**
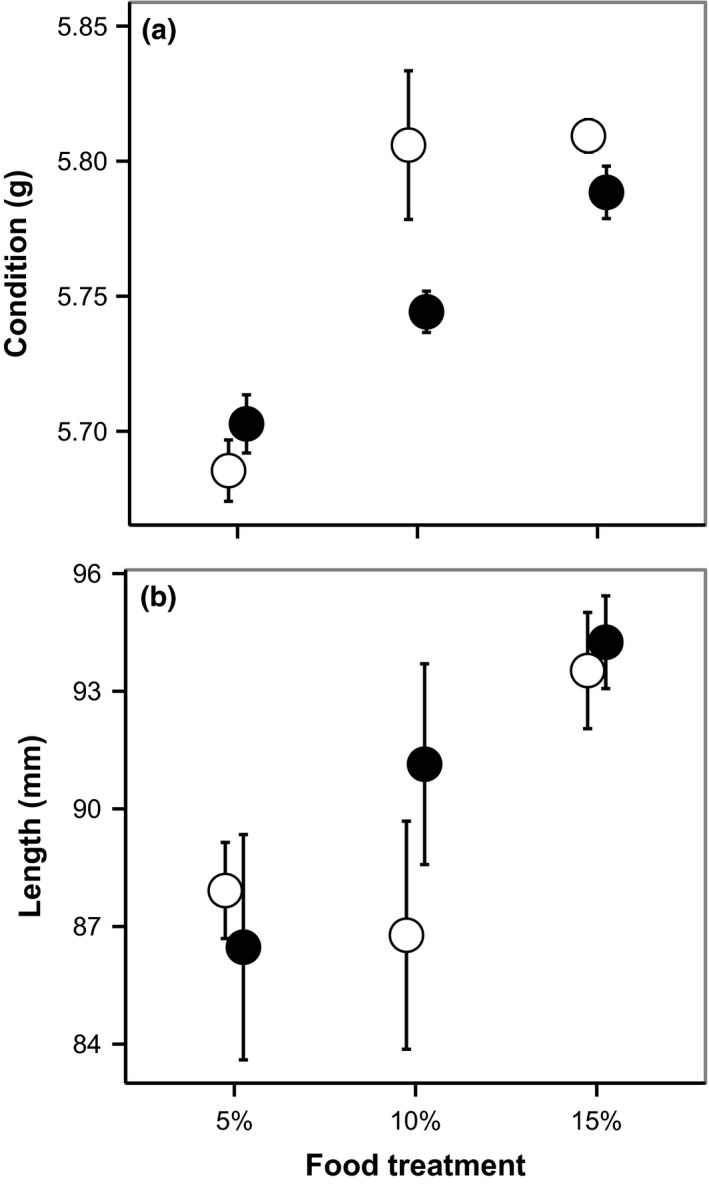
Effects of treatments (food and pike) on (a) condition and (b) length of perch at the end of the experiment. Condition was calculated as the estimated weight for a 90‐mm‐long individual; see methods for more details. Filled symbols are treatments with pike; open symbols are treatments without pike. Data are means ± *SE*

With an increase in food level, the condition of the perch increased whereas the effect of predation was marginally insignificant (Figure 1a, Table 1). Notably, we also found a significant predation–food level interaction on condition (Figure 1a, Table 1). Post hoc tests revealed a relatively lower condition of perch in the presence of predator at the 10% food level (Tukey, *p* = .006), whereas there was no effect of predator presence at either the 5% (Tukey, *p* = .357) or 15% (Tukey, *p* = .283) food levels. The increased food level also resulted in longer individuals (Figure 1b, Table 1), whereas neither predation nor interaction effect was found on length.

**Table 1:** Results from nested ANOVA on condition and total length at the end of the experiment

	*df*	Condition		Length	
		*F*‐value	*p*‐Value	*F*‐value	*p*‐Value
Predator	1,30	3.60	.066	0.724	.402
Food	2,30	**30.91**	**<.001**	**5.211**	**.011**
Pred. × Food	2,30	**3.73**	**.037**	0.486	.620

Significant effects are highlighted in bold.

### 3.2 Behavior

**Figure 2:**
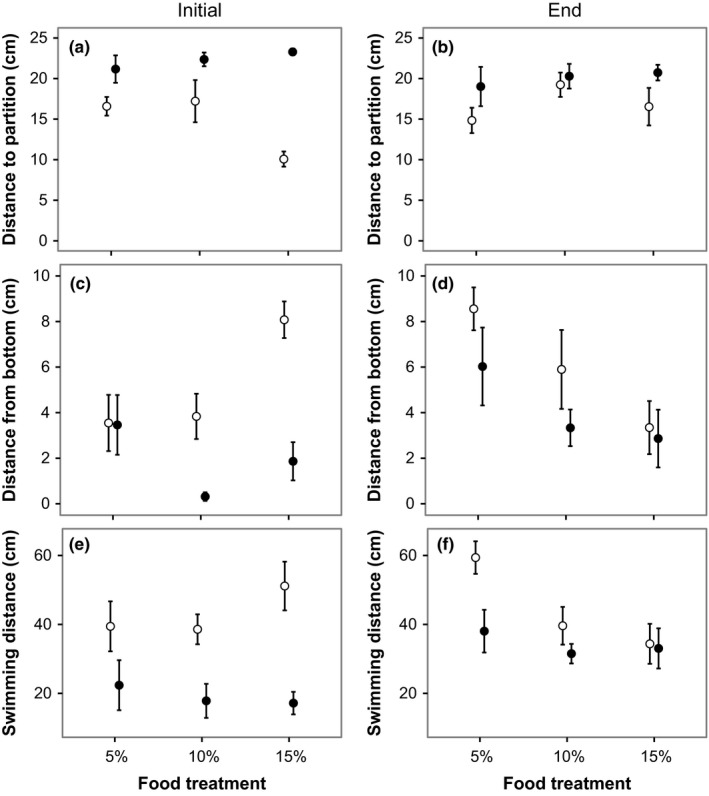
Initial (left panel) behavioral responses and behavioral responses at the end of the experiment (right panel) for the food and pike treatments. (a) and (b) are the distance from the partitioning that separates the focal perch from the pike (in the pike treatment), (c) and (d) are distance from the bottom of the tank, and (e) and (f) are the distance swam per minute. Filled symbols are treatments with pike; open symbols are treatments without pike. Data are means ± *SE*

Both at the start and at the end of the experiment, perch in treatments with predators were further away from the predator partition compared to perch without predators (Figure 2a,b, Table 2a). At the start of the experiment, there was a significant predator–food level interaction because perch were further away from the partition in the presence than in the absence of predator in the 15% food‐level treatment (post hoc, Tukey, *p* < .001) but not in the 10% (Tukey, *p* = .175) and 5% (Tukey, *p* = .271) food levels.

**Table 2:** Results from nested ANOVA on behavior at the start and the end of the experiment for (a) distance from predator, (b) distance from bottom, and (c) swimming distance during 1 min

	*df*	Start of experiment		End of experiment	
		*F*‐value	*p*‐Value	*F*‐value	*p*‐Value
(a) Distance from predator					
Predator	1,30	**40.006**	**<.001**	**5.065**	**.032**
Food	2,30	2.401	.108	1.388	.265
Pred. × Food	2,30	**5.362**	**.010**	0.650	.529
(b) Distance from bottom					
Predator	1,30	**16.833**	**<.001**	2.958	.096
Food	2,30	**4.501**	**.020**	**4.810**	**.015**
Pred. × Food	2,30	**5.154**	**.012**	0.412	.666
(c) Swimming distance					
Predator	1,30	**23.577**	**<.001**	**5.110**	**.031**
Food	2,30	0.535	.591	**4.105**	**.027**
Pred. × Food	2,30	1.253	.300	1.632	.212

Significant effects are highlighted in bold.

The perch also spent more time closer to the bottom in the predator treatments at the start but not at the end of the experiment (Figure 2c,d, Table 2b). In addition, at the beginning and at the end, there was an effect of food‐level treatment on vertical position (Figure 2c,d, Table 2b). At the start of the experiment, perch spent more time high up in the water column in the higher food‐level treatments, while the opposite pattern was found at the end of the experiment (Figure 2c,d). Furthermore, at the start of the experiment, there was a significant predator–food level interaction suggesting that the effect of food level was caused by perch in high food‐level treatments spending more time up in the water column in the absence of predator compared to in the presence of predator (Figure 2c, post hoc effect of predator treatment, 5%: Tukey, *p* = .985, 10%: *p* = .016, 15%: *p* < .001).

Perch reduced their activity in the presence of predators both at the start and at the end of the experiment (Figure 2e,f, Table 2c). The reduced activity at the end of the experiment in response to food level (Figure 2f) was because of a difference in activity between the 5% and 10% food ration treatments (Tukey, *p* = .029) and the 5% and 15% food ration treatments (Tukey, *p* = .005), whereas no difference was found between the 10% and 15% food ration treatments (Tukey, *p* = .790).

### 3.3 Body shape

**Figure 3:**
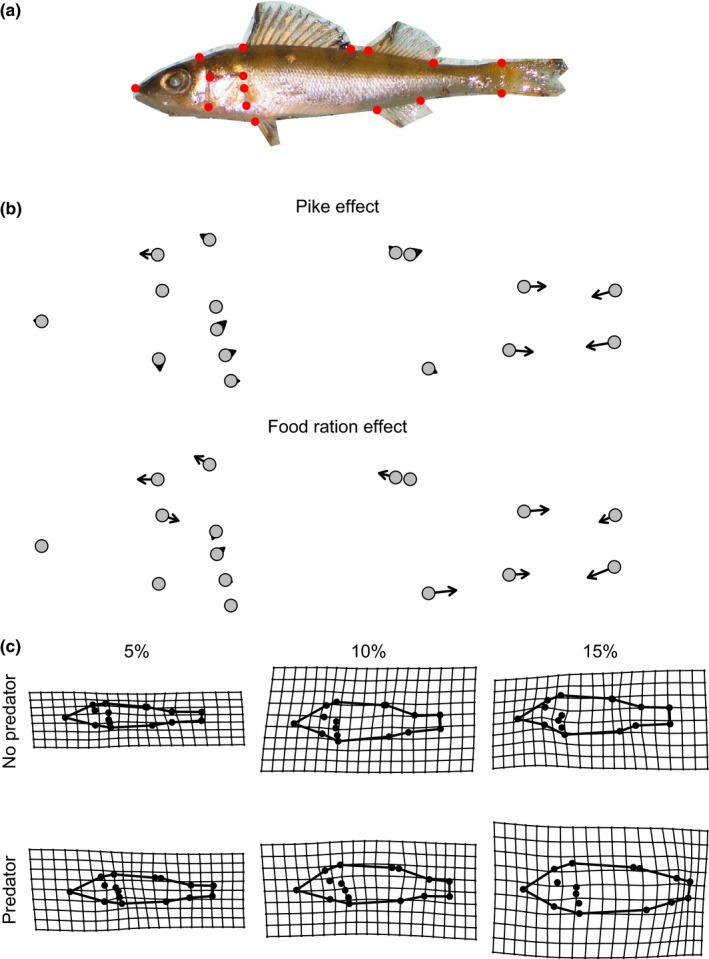
Visualizations of landmarks and general shape changes as a response to pike and food treatment in perch. (a) Position of landmarks used for analyzing morphological plasticity in this study. (b) Illustrations of the main effects of pike and food treatments showing displacements (vectors) of landmarks in response to treatments. (c) Grid plots showing mean shape in the different treatment combinations

The MANCOVA on the first four shape variables (principal components) showed that there were effects of both predation and food level on perch body shape as well as all activity, size, and condition (Table 3). Overall, perch in treatments with predators developed a deeper body shape compared to perch without predators (Figure 3). Similarly, perch in higher food‐level treatments also developed deeper body shapes (Figure 3). The majority of the vectors changed in the same direction from low to high food as for no predator to predator (Figure 3b). There was also a significant interaction between predation and food level on perch shape, suggesting that the difference in shape between predator and no‐predator treatment increased with increasing food‐level treatment (Figure 3C).

**Table 3:** Results from MANCOVA on shape using activity levels in the beginning and at the end of the experiment as well as the centroid size and condition as covariates

	*df*	Wilks λ	*F*‐value	*p*‐Value
Predator	1,26	**0.30378**	**13.179**	**<.001**
Food	2,26	**0.29002**	**4.927**	**<.001**
Pred. × Food	2,26	**0.43714**	**2.947**	**.010**
Activity beginning	1,26	**0.4103**	**8.265**	**<.001**
Activityend	1,26	**0.29328**	**13.856**	**<.001**
Size	1,26	**0.42536**	**7.768**	**<.001**
Condition	1,26	**0.19560**	**23.646**	**<.001**

Significant effects are highlighted in bold.

**Figure 4:**
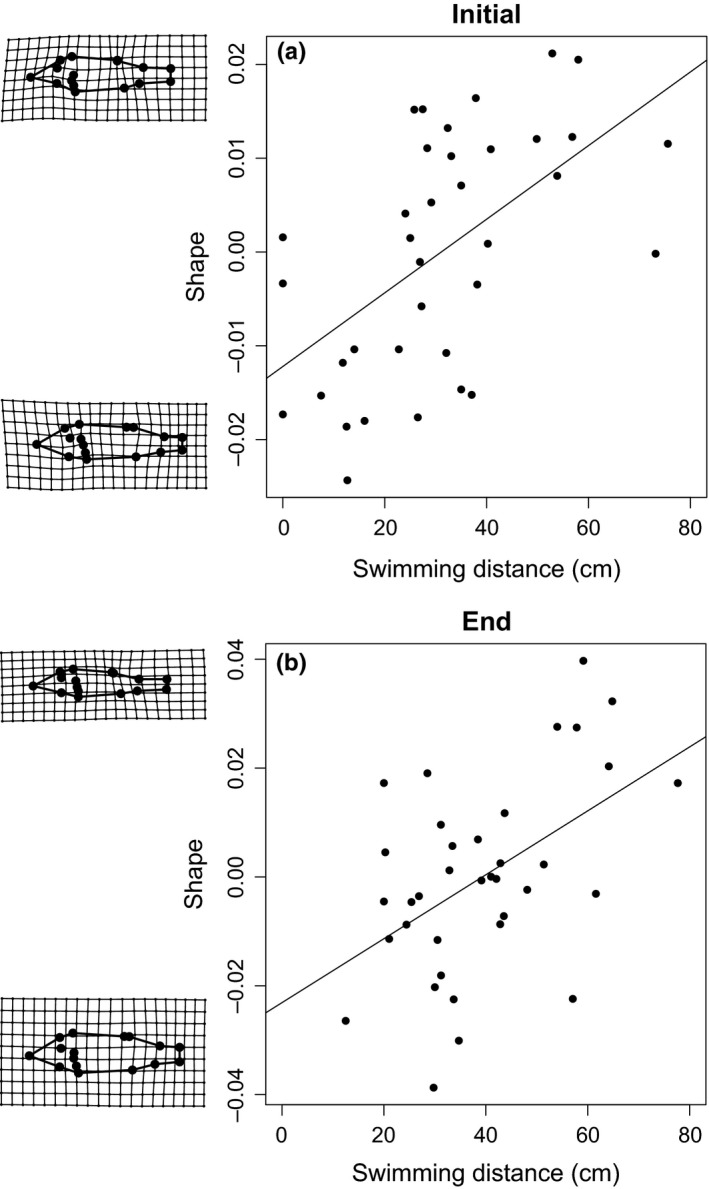
Correlation between shape and activity over all treatments at (a) the start of the experiment and (b) the end of the experiment. Grid plots to the left of the graphs illustrate shape changes from negative to positive scores and are exaggerated six times to facilitate interpretations. Data points represent tank (replicate) averages of shape and activity

**Figure 5:**
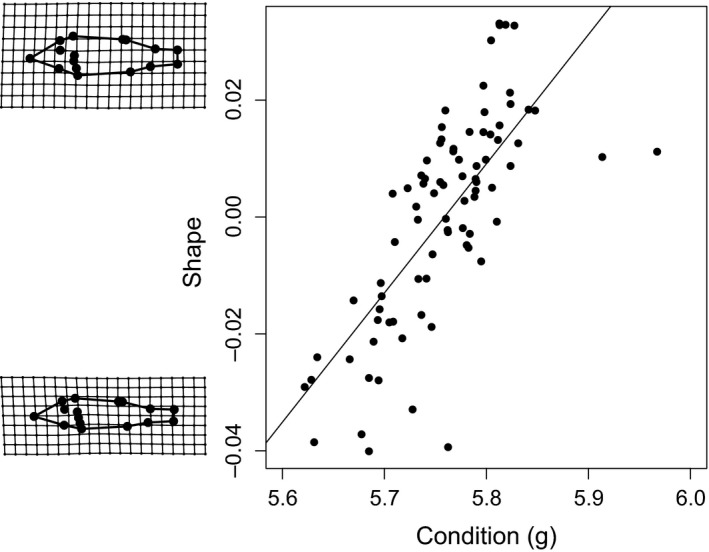
Correlation between condition and shape. Grid plots to the left of the graphs illustrate shape changes from negative to positive scores and are exaggerated two times to facilitate interpretations. We estimated condition of each individual by first performing a regression on all log‐transformed lengths and weights and saving the residuals. From this regression, we calculated the weight for an average individual of 90 mm. Condition for each fish was then estimated as the calculated average weight plus the residual

The effect of activity was stronger for the activity measures at the end of the experiment (Table 3), but in general, more active fish had longer caudal peduncles and were generally more streamlined than nonactive fish (Figure 4). Further, there was also a significant effect of both body condition and centroid size on body shape (Table 3), where fish in better condition was deeper bodied than fish in poor condition (Figure 5) and larger fish were deeper bodied than smaller fish.

**Figure 6:**
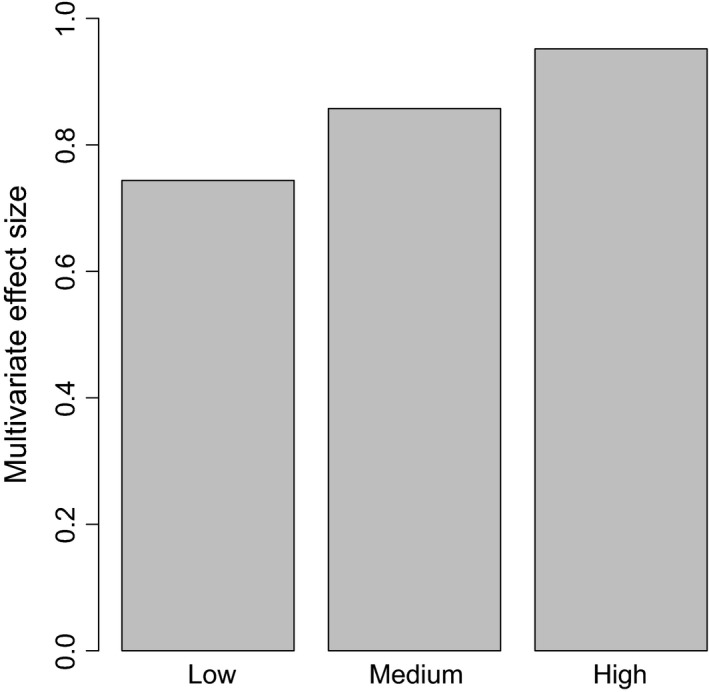
Divergence in morphology (multivariate effect size) between the pike and no‐pike treatments for the different food levels. A larger value indicates a larger morphological divergence

The analysis on effect size differences between food‐level treatments on the effect of predation showed that the multivariate effect size increased with increasing food level, that is, the effect of predation on morphological plasticity was higher in treatments with higher food levels (Figure 6). When analyzing the predation effects at each food ratio treatment, we found significant effects of predator presence in the high food‐level treatment (MANOVA; Wilks λ = 0.048, *F*_5,6_ = 23.69, *p* < .001) as well as in the medium food‐level treatment (MANOVA; Wilks λ = 0.143, *F*_5,6_ = 7.23, *p* = .016) but not in the low food‐level treatment (MANOVA; Wilks λ = 0.256, *F*_5,6_ = 3.48, *p* = .080).

**Figure 7:**
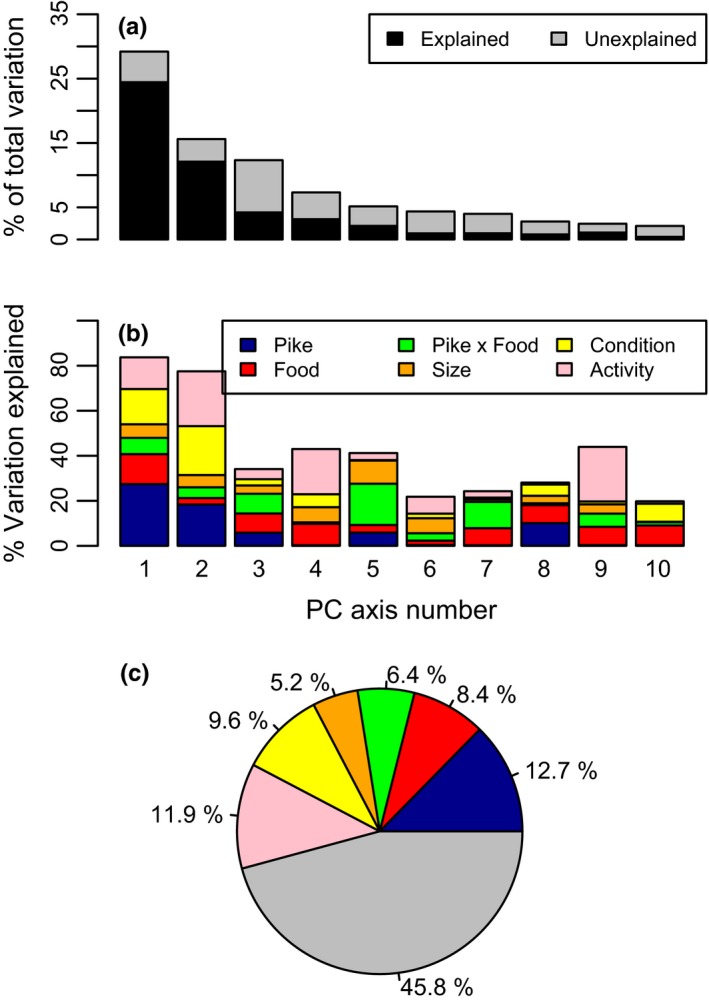
Estimates of the relative effect sizes on morphology in perch in the experiment. We used the calculated PC axes, as quantitative axes summarizing among individual variation in morphology. (a) The percentage of the total morphological variation explained by each PC axes 1 through 10 (height of each bar). For each PC axis, we used an ANCOVA framework to estimate the percent variation explained by our treatments (food, pike, and the interaction food × pike), size (centroid), condition, as well as our behavioral measurements (both after 1 week and at the end of the experiment). The black portion of each PC axis bar represents the variation explained by the linear model (gray shading indicates unexplained residual variation). (b) The percent of each PC's variation explained by food, pike, the interaction food × pike, size, condition, and behavior. (c) Summing the percent variation explained by each factor, across all PC axes (not just the 10 shown here), weighted by the % of total variation attributed to each PC axis

To explore the relative importance of all our treatments and analyzed variables on body shape, we used linear models to simultaneously estimate the effect size (% variance explained) of treatments (food, predator, and the interaction food × predator), size (centroid), condition, as well as behavior (swimming activity; both after 1 week and at the end of the experiment) on the PC axes explaining body shape. We found that predator treatment explained 12.7%, followed by activity 11.9%, body condition (9.6%), food treatment (8.4%), the interaction food × predator 6.4%, and centroid size (5.2%) (Figure 7).

## 4 DISCUSSION

Phenotypic plasticity of body shape in fishes can be affected by multiple factors (Ab Ghani, Herczeg, & Merila, 2016; Andersson et al., 2006; Holopainen et al., 1997; Langerhans, Layman, Shokrollahi, & DeWitt, 2004; Olsson et al., 2006). Here we showed that the presence of a predator, food level, and behavior affected body shape of perch in an additive and interactive way by inducing a deeper body shape, with predation and behavior having the largest and food treatment the smallest effect. These shape changes seemed to be mediated by changes in growth rate, as body condition caused a similar effect to shape as our other factors (Figure 7). A deeper body has an adaptive value in the presence of predators as deeper bodied individuals enforce a longer handling time for the predator or may even exclude gape limited predators from feeding on the prey (Nilsson et al., 1995), but we note that a similar body shape is also induced by food level and behavior.

### 4.1 Predator effect

Predators have been shown to induce body shape change in many aquatic animals (Boersma et al., 1998; Tollrian & Harvell, 1999). In fish, several studies have shown that the presence of predators induces a change in body shape (Brönmark & Miner, 1992; Eklöv & Jonsson, 2007; Langerhans & DeWitt, 2004; Langerhans et al., 2004). For perch, we have here shown that the presence of predators induces a deepening of the body, especially in the caudal region. This may be an adaptive plastic response to predators as having a deeper mid‐body/caudal region maximizes burst‐swimming performance, which is essential for escaping predator attack (Domenici, Turesson, Brodersen, & Brönmark, 2008; Taylor & McPhail, 1986). In addition, deeper bodies may also reduce the risk of predation as it increases handling time of predators (Nilsson et al., 1995). The more streamlined shape in the no‐predator treatment may be an adaptive plastic response to steady‐swimming performance (Domenici et al., 2008; Webb, 1984).

### 4.2 Food effect

The food‐level effect on shape in perch explained 8.4% of the variation in perch shape in this experiment. Similar results were found for crucian carp (Brönmark & Miner, 1992) and goldfish (Chivers et al., 2008), where increased food levels resulted in a significantly deeper body shape. The food level also had a strong influence on perch growth rate in the experiment. It is thus likely that the differences in growth rate induced by food level also affected the shape of perch. In addition to this, the amount of food also affected body condition in perch. Not surprisingly, body condition was directly related to shape where 9.6% of the total shape variation was explained by condition. Other studies have also found that body condition affects body shape in fish (Holopainen et al., 1997; Olsson et al., 2006). Our results suggest that there is a strong covariation between shape and condition in fish. This may be because our measure of condition (estimated weight at 90 mm) may reflect the bulkiness of the individual and thereby be directly related to the body depth of the individual. However, this covariation with shape may depend on the chosen method to estimate condition.

Notably, we found that food level and predation interacted in affecting body shape in perch as well as perch condition. We observed the strongest effect of predators on body shape at the highest food level. This may indicate a production cost of the plastic phenotype in perch. It also suggests that reduced food availability and, hence, decreased growth rate could reduce the potential for plasticity‐driven morphological divergence in response to predators (Andersson et al., 2006). This has also been suggested as the mechanism behind plasticity‐driven morphological divergence between habitats (Olsson et al., 2006, 2007). To date, we do not know the underlying physiological mechanism behind the changes in body shape. However, taken together with a previous study on perch (Olsson et al., 2006, 2007), the results presented here clearly indicate that growth rate might play a key role in regulating morphological differentiation in perch. A tentative mechanism is that at low growth rates, the energy gained by foraging is mainly used for metabolic maintenance, whereas at higher growth rates a surplus of energy is available which can be allocated to tissue growth and thus morphological modulation (Holopainen et al., 1997; Post & Parkinson, 2001). Whether more cells or larger cells per unit of body tissues drive this and which genes that are associated with these changes awaits future research.

### 4.3 Indirect effects of activity

Behavioral differences have been postulated to precede morphological transitions during the process of diversification (Price, Qvarnström, & Irwin, 2003; West‐Eberhard, 1989). In this sense, it has been suggested that body shape induced by the environment may be indirectly mediated by differences in behavior (Bourdeau & Johansson, 2012). For example, prey can reduce their activity, and thus metabolism, in the presence of predators, which may save energy (Holopainen et al., 1997; Scheiner & Berrigan, 1998; Steiner & Van Buskirk, 2009) that could be used for an increase in growth rate, which ultimately could affect body shape (Olsson et al., 2006). Also, many animals reduce activity at higher food levels (Relyea, 2004; Werner & Anholt, 1993), and the reduced activity might thus reduce energy use (McPeek, 1995; Webb, 1984) which in turn could affect individual morphology through an increased availability of energy for building the plastic phenotype. An exposure to different resource levels or the presence of predators could thus result in a behaviorally mediated response in morphology (Relyea, 2004).

In this study, we found that both presence of a predator and food level affected behavior and growth in perch. The responses in behavior were in the direction as expected. First, the presence of predators reduced activity and distance to predators and this would decrease predation risk in a natural setting (Johansson, Englund, Brodin, & Gardfjell, 2006). Second, fish at high food levels decreased activity and increased distance in the response to the presence of predators more than fish at low food levels, suggesting that fish at high food levels can afford lower foraging activity, thereby reducing predation risk (Godin & Smith, 1988). The reduced activity level in the presence of predators saves energy and, hence, has the potential to increase growth rate, and thereby body depth, as observed. The effect size suggested that the effect of behavior on body shape was similar to that of predation risk. Taken together, our results indicate that body shape was affected by a combination of induced activity changes and our food and predator treatments. We also note that as both our food and predator treatments affected activity, the overall effects of food and predation may be larger if the indirect path through behavior would be accounted for. However, our experimental design does not allow us to connect an individual's behavior to its morphology and hence precludes such an analysis.

### 4.4 Behavioral differences over time

Overall, we had similar behavioral responses to our treatments both in the beginning and at the end of the experiment, but there were also some notable changes over time in the response to our treatments. In our analyses, we found that both initial activity and the activity at the end influenced shape, where the effect of activity at the end had a slightly stronger response. The change in time of the behavioral responses could be due to several reasons. First, it could be a result of habituation to predation risk, such that after a couple of weeks the perch actually do not perceive the predators as a risk (Ferrari, Sih, & Chivers, 2009). However, in nature, prey fish might be subjected to spatial as well as temporal variation in predation risk and hence predation risk would vary over time. Therefore, habituation might be of little importance under natural conditions. Second, it could be an effect of decreased risk as perch grow. At the end of the experiment, the low food‐level fish still showed a behavioral response while the high food‐level fish showed little or no antipredator response. Perhaps the larger fish at the high food level had grown to a size/shape where predation risk is reduced. It would be interesting to see how this would further influence the phenotype of the fish.

Typically, laboratory experiments on body shape in fish are run for 8–10 weeks (Andersson et al., 2006; Brönmark & Miner, 1992; Holopainen et al., 1997) and it remains an open question how body shape and behavior vary and develop over longer time in experiments that manipulate temporal variation in predation risk (see Chivers et al., 2008). Nevertheless, field data and field experiments show that fish in the presence of predators or at high per capita food resources show body shape changes in the same direction as in these short term laboratory experiments (Brönmark & Miner, 1992; Holopainen et al., 1997). In the future, it would be interesting to explore population and community ecology‐level effects of body shape differences under natural conditions in the wild.

### 4.5 Conclusion

Here we have focused on a freshwater system to study how predation, resource levels, and behavior affect body shape. Studies on how body shape in prey organisms is affected by predation and resource levels are biased toward freshwater and marine environments (Bourdeau, 2012; Brönmark, Lakowitz, & Hollander, 2011; Langerhans et al., 2004; Nunes, Orizaola, Laurila, & Rebelo, 2014). That does not mean that these effects do not occur in terrestrial systems, but traditionally they have been studied with traditional linear morphometric methods in terrestrial environments (Bula, Wright, & Zani, 2014; Losos, Schoener, Langerhans, & Spiller, 2006). Therefore, a comparison with nonaquatic system is difficult to make with regard to general results on body shape. Nevertheless, our results suggest that not only direct effects of our treatments, but also indirect effects through behavior affected body shape in perch. Although we found a general increase in body depth in response to predation risk, higher food levels, and reduced activity, we note that all three factors affected different aspects of changes in perch body morphology at a finer body shape scale. Our results show that the regulation of plastic responses in body shape of vertebrates is a complex process in need of further research. Especially important would be to disentangle how the induced shapes affect predator avoidance and foraging efficiencies.

## CONFLICT OF INTEREST

None decaled.

## DATA ACCESSIBILITY

Data for this article are deposited in the Dryad Digital Repository: doi:10.5061/dryad.975m1.

## AUTHOR CONTRIBUTION

RS, YZ, CB, and FJ conceived the project. YZ implemented the experiment. RS analyzed the data and wrote the manuscript with help from all coauthors.

## References

[ece33330-bib-0001] Ab Ghani, N. I. , Herczeg, G. , & Merila, J. (2016). Effects of perceived predation risk and social environment on the development of three‐spined stickleback (*Gasterosteus aculeatus*) morphology. Biological Journal of the Linnean Society, 118, 520–535. https://doi.org/10.1111/bij.12783

[ece33330-bib-0002] Adams, D. C. , & Otarola‐Castillo, E. (2013). geomorph: An r package for the collection and analysis of geometric morphometric shape data. Methods in Ecology and Evolution, 4, 393–399. https://doi.org/10.1111/2041-210x.12035

[ece33330-bib-0003] Andersson, J. , Johansson, F. , & Söderlund, T. (2006). Interactions between predator‐ and diet‐induced phenotypic changes in body shape of crucian carp. Proceedings of the Royal Society B‐Biological Sciences, 273, 431–437. https://doi.org/10.1098/rspb.2005.3343 10.1098/rspb.2005.3343 PMC1560211 16615209

[ece33330-bib-0004] Arnqvist, G. , & Johansson, F. (1998). Ontogenetic reaction norms of predator‐induced defensive morphology in dragonfly larvae. Ecology, 79, 1847–1858.

[ece33330-bib-0005] Benkman, C. W. , & Lindholm, A. K. (1991). The advantages and evolution of a morphological novelty. Nature, 349, 519–520. https://doi.org/10.1038/349519a0

[ece33330-bib-0006] Björklund, M. , & Merila, J. (1993). Morphological‐differentiation in Carduelis finches ‐ adaptive vs constraint models. Journal of Evolutionary Biology, 6, 359–373.

[ece33330-bib-0007] Boersma, M. , Spaak, P. , & De Meester, L. (1998). Predator‐mediated plasticity in morphology, life history, and behavior of Daphnia: The uncoupling of responses. American Naturalist, 152, 237–248. https://doi.org/10.1086/286164 10.1086/286164 18811388

[ece33330-bib-0008] Bourdeau, P. E. (2012). Intraspecific trait cospecialization of constitutive and inducible morphological defences in a marine snail from habitats with different predation risk. Journal of Animal Ecology, 81, 849–858. https://doi.org/10.1111/j.1365-2656.2012.01965.x 22320427 10.1111/j.1365-2656.2012.01965.x

[ece33330-bib-0009] Bourdeau, P. E. , & Johansson, F. (2012). Predator‐induced morphological defences as by‐products of prey behaviour: A review and prospectus. Oikos, 121, 1175–1190. https://doi.org/10.1111/j.1600-0706.2012.20235.x

[ece33330-bib-0010] Brönmark, C. , Lakowitz, T. , & Hollander, J. (2011). Predator‐induced morphological plasticity across local populations of a freshwater snail. PLoS ONE, 6, 6 https://doi.org/10.1371/journal.pone.0021773 10.1371/journal.pone.0021773 PMC3139574 21818264

[ece33330-bib-0011] Brönmark, C. , & Miner, J. G. (1992). Predator‐induced phenotypical change in body morphology in Crucian carp. Science, 258, 1348–1350. 17778362 10.1126/science.258.5086.1348

[ece33330-bib-0012] Bula, P. A. , Wright, L. K. , & Zani, P. A. (2014). Geographic variation in lizard hind‐limb morphology in relation to predation: No evidence for an evolutionary basis. Evolutionary Ecology Research, 16, 663–687.

[ece33330-bib-0013] Chivers, D. P. , Zhao, X. X. , Brown, G. E. , Marchant, T. A. , & Ferrari, M. C. O. (2008). Predator‐induced changes in morphology of a prey fish: The effects of food level and temporal frequency of predation risk. Evolutionary Ecology, 22, 561–574. https://doi.org/10.1007/s10682-007-9182-8

[ece33330-bib-0014] Dayton, G. H. , Saenz, D. , Baum, K. A. , Langerhans, R. B. , & DeWitt, T. J. (2005). Body shape, burst speed and escape behavior of larval anurans. Oikos, 111, 582–591.

[ece33330-bib-0015] DeWitt, T. J. , & Scheiner, S. M. (2004). Phenotypic plasticity ‐ functional and conceptual approaches. Oxford: Oxford University Press.

[ece33330-bib-0016] Domenici, P. , Turesson, H. , Brodersen, J. , & Brönmark, C. (2008). Predator‐induced morphology enhances escape locomotion in crucian carp. Proceedings of the Royal Society B‐Biological Sciences, 275, 195–201. https://doi.org/10.1098/rspb.2007.1088 10.1098/rspb.2007.1088 PMC2596180 17971327

[ece33330-bib-0017] Dryden, I. L. (2015). Shapes package. Vienna, Austria: R Foundation for Statistical Computing Contributed package. Version 1‐1.11. Retrieved from http://www.R-project.org

[ece33330-bib-0018] Eklöv, P. , & Jonsson, P. (2007). Pike predators induce morphological changes in young perch and roach. Journal of Fish Biology, 70, 155–164. https://doi.org/10.1111/j.1095-8649.2006.01283.x

[ece33330-bib-0019] Eklöv, P. , & Persson, L. (1995). Species‐specific antipredator capacities and prey refuges ‐ interactions between piscivorous perch (*Perca‐fluviatilis*) and Juvenile Perch and Roach (*Rutilus‐rutilus*). Behavioral Ecology and Sociobiology, 37, 169–178.

[ece33330-bib-0020] Eklöv, P. , & Persson, L. (1996). The response of prey to the risk of predation: Proximate cues for refuging juvenile fish. Animal Behaviour, 51, 105–115.

[ece33330-bib-0021] Eklöv, P. , & Svanbäck, R. (2006). Predation risk influences adaptive morphological variation in fish populations. American Naturalist, 167, 440–452. 10.1086/499544 16673351

[ece33330-bib-0022] Ferrari, M. C. O. , Sih, A. , & Chivers, D. P. (2009). The paradox of risk allocation: A review and prospectus. Animal Behaviour, 78, 579–585. https://doi.org/10.1016/j.anbehav.2009.05.034

[ece33330-bib-0023] Godin, J. G. J. , & Smith, S. A. (1988). A fitness cost of foraging in the guppy. Nature, 333, 69–71. https://doi.org/10.1038/333069a0

[ece33330-bib-0024] Holopainen, I. J. , Aho, J. , Vornanen, M. , & Huuskonen, H. (1997). Phenotypic plasticity and predator effects on morphology and physiology of crucian carp in nature and in the laboratory. Journal of Fish Biology, 50, 781–798.

[ece33330-bib-0025] Johansson, F. , Englund, G. , Brodin, T. , & Gardfjell, H. (2006). Species abundance models and patterns in dragonfly communities: Effects of fish predators. Oikos, 114, 27–36. https://doi.org/10.1111/j.2006.0030-1299.14495.x

[ece33330-bib-0026] Krist, A. C. (2002). Crayfish induce a defensive shell shape in a freshwater snail. Invertebrate Biology, 121, 235–242.

[ece33330-bib-0027] Langerhans, R. B. , & DeWitt, T. J. (2004). Shared and unique features of evolutionary diversification. American Naturalist, 164, 335–349. https://doi.org/10.1086/422857 10.1086/422857 15478089

[ece33330-bib-0028] Langerhans, R. B. , Layman, C. A. , Shokrollahi, A. M. , & DeWitt, T. J. (2004). Predator‐driven phenotypic diversification in *Gambusia affinis* . Evolution, 58, 2305–2318. 15562692 10.1111/j.0014-3820.2004.tb01605.x

[ece33330-bib-0029] Lessmark, O. (1983). Competition between perch (Perca fluviatilis) and roach (Rutilus rutilus) in south Swedish lakes. PhD, University of Lund, Lund.

[ece33330-bib-0030] Losos, J. B. (1990). The evolution of form and function ‐ morphology and locomotor performance in West‐Indian anolis lizards. Evolution, 44, 1189–1203. https://doi.org/10.2307/2409282 28563896 10.1111/j.1558-5646.1990.tb05225.x

[ece33330-bib-0031] Losos, J. B. , Schoener, T. W. , Langerhans, R. B. , & Spiller, D. A. (2006). Rapid temporal reversal in predator‐driven natural selection. Science, 314, 1111 https://doi.org/10.1126/science.1133584 17110568 10.1126/science.1133584

[ece33330-bib-0032] McDonald, J. H. (2014). Handbook of biological statistics, 3rd ed Baltimore, MD: Sparky House Publishing.

[ece33330-bib-0033] McPeek, M. A. (1995). Morphological evolution mediated by behavior in the damselflies of 2 communities. Evolution, 49, 749–769. 28565148 10.1111/j.1558-5646.1995.tb02311.x

[ece33330-bib-0034] Newman, R. A. (1992). Adaptive plasticity in amphibian metamorphosis. BioScience, 42, 671–678.

[ece33330-bib-0035] Nilsson, P. A. , Brönmark, C. , & Pettersson, L. B. (1995). Benefits of a predator‐induced morphology in crucian carp. Oecologia, 104, 291–296. https://doi.org/10.1007/bf00328363 28307584 10.1007/BF00328363

[ece33330-bib-0036] Nunes, A. L. , Orizaola, G. , Laurila, A. , & Rebelo, R. (2014). Morphological and life‐history responses of anurans to predation by an invasive crayfish: An integrative approach. Ecology and Evolution, 4, 1491–1503. https://doi.org/10.1002/ece3.979 24834343 10.1002/ece3.979 PMC4020706

[ece33330-bib-0037] Olsson, J. , Svanbäck, R. , & Eklöv, P. (2006). Growth rate constrain morphological divergence when driven by competition. Oikos, 115, 15–22.

[ece33330-bib-0038] Olsson, J. , Svanbäck, R. , & Eklöv, P. (2007). Effects of resource level and habitat type on behavioral and morphological plasticity in Eurasian perch. Oecologia, 152, 48–56. 17431684 10.1007/s00442-006-0588-8

[ece33330-bib-0039] Persson, L. , Byström, P. , & Wahlström, E. (2000). Cannibalism and competition in Eurasian perch: Population dynamics of an ontogenetic omnivore. Ecology, 81, 1058–1071.

[ece33330-bib-0040] Pettersson, L. B. , & Brönmark, C. (1997). Density‐dependent costs of an inducible morphological defense in crucian carp. Ecology, 78, 1805–1815.

[ece33330-bib-0041] Post, J. R. , & Parkinson, E. A. (2001). Energy allocation strategy in young fish: Allometry and survival. Ecology, 82, 1040–1051.

[ece33330-bib-0042] Price, T. D. , Qvarnström, A. , & Irwin, D. E. (2003). The role of phenotypic plasticity in driving genetic evolution. Proceedings of the Royal Society of London Series B‐Biological Sciences, 270, 1433–1440. 10.1098/rspb.2003.2372 PMC1691402 12965006

[ece33330-bib-0043] R Development Core Team (2013). R: A language and environment for statistical computing. Vienna, Austria: R Foundation for Statistical Computing.

[ece33330-bib-0044] Relyea, R. A. (2004). Fine‐tuned phenotypes: Tadpole plasticity under 16 combinations of predators and competitors. Ecology, 85, 172–179. https://doi.org/10.1890/03-0169

[ece33330-bib-0045] Scheiner, S. M. (1993). Genetics and evolution of phenotypic plasticity. Annual Review of Ecology and Systematics, 24, 35–68.

[ece33330-bib-0046] Scheiner, S. M. , & Berrigan, D. (1998). The genetics of phenotypic plasticity. VIII. The cost of plasticity in *Daphnia pulex* . Evolution, 52, 368–378. 28568340 10.1111/j.1558-5646.1998.tb01638.x

[ece33330-bib-0047] Sharma, S. (1996). Applied multivariate techniques. New York, NY: John Wiley & Sons.

[ece33330-bib-0048] Stauffer, J. R. , & Gray, E. V. (2004). Phenotypic plasticity: Its role in trophic radiation and explosive speciation in cichlids (Teleostei: Cichlidae). Animal Biology, 54, 137–158.

[ece33330-bib-0049] Steiner, U. K. (2007). Investment in defense and cost of predator‐induced defense along a resource gradient. Oecologia, 152, 201–210. https://doi.org/10.1007/s00442-006-0645-3 17221255 10.1007/s00442-006-0645-3

[ece33330-bib-0050] Steiner, U. K. , & Van Buskirk, J. (2009). Predator‐induced changes in metabolism cannot explain the growth/predation risk tradeoff. PLoS ONE. https://doi.org/e616010.1371/journal.pone.0006160 10.1371/journal.pone.0006160 PMC2701611 19582147

[ece33330-bib-0051] Svanbäck, R. , & Eklöv, P. (2003). Morphology dependent foraging efficiency in perch: A trade‐off for ecological specialization? Oikos, 102, 273–284.

[ece33330-bib-0052] Taylor, E. B. , & McPhail, J. D. (1986). Prolonged and burst swimming in anadromous and fresh‐water threespine stickleback, *Gasterosteus aculeatus* . Canadian Journal of Zoology‐Revue Canadienne De Zoologie, 64, 416–420. https://doi.org/10.1139/z86-064

[ece33330-bib-0053] Teplitsky, C. , & Laurila, A. (2007). Flexible defense strategies: Competition modifies investment in behavioral vs. morphological defenses. Ecology, 88, 1641–1646. https://doi.org/10.1890/06-1703.1 17645010 10.1890/06-1703.1

[ece33330-bib-0054] Tollrian, R. , & Harvell, C. D. (1999). The ecology and evolution of inducible defenses. Princeton, NJ: Princeton University Press.

[ece33330-bib-0055] Tonn, W. M. , Holopainen, I. J. , & Paszkowski, C. A. (1994). Density‐dependent effects and the regulation of crucian carp populations in single‐species ponds. Ecology, 75, 824–834.

[ece33330-bib-0056] Vollestad, L. A. , Varreng, K. , & Poleo, A. B. S. (2004). Body depth variation in crucian carp *Carassius carassius*: An experimental individual‐based study. Ecology of Freshwater Fish, 13, 197–202. https://doi.org/10.1111/j.1600-0633.2004.00048.x

[ece33330-bib-0057] Webb, P. W. (1984). Body form, locomotion and foraging in aquatic vertebrates. American Zoologist, 24, 107–120.

[ece33330-bib-0058] Werner, E. E. , & Anholt, B. R. (1993). Ecological consequences of the trade‐off between growth and mortality‐rates mediated by foraging activity. American Naturalist, 142, 242–272. 10.1086/285537 19425978

[ece33330-bib-0059] West‐Eberhard, M. J. (1989). Phenotypic plasticity and the origins of diversity. Annual Review of Ecology and Systematics, 20, 249–278.

[ece33330-bib-0060] Zelditch, M. L. , Swiderski, D. L. , Sheets, H. D. , & Fink, W. L. (2004). Geometric morphometrics for biologists. Burlington, Massachusetts, USA: Elsevier Academic Press.

